# Transcriptional analysis of adipose tissue during development reveals depot-specific responsiveness to maternal dietary supplementation

**DOI:** 10.1038/s41598-018-27376-3

**Published:** 2018-06-25

**Authors:** Hernan P. Fainberg, Mark Birtwistle, Reham Alagal, Ahmad Alhaddad, Mark Pope, Graeme Davies, Rachel Woods, Marcos Castellanos, Sean T. May, Catharine A. Ortori, David A. Barrett, Viv Perry, Frank Wiens, Bernd Stahl, Eline van der Beek, Harold Sacks, Helen Budge, Michael E. Symonds

**Affiliations:** 1 Division of Child Health, Obstetrics & Gynaecology, The University of Nottingham, Nottingham, United Kingdom; 2 Nottingham Digestive Disease Centre and Biomedical Research Centre, School of Medicine, Queen’s Medical Centre, The University of Nottingham, Nottingham, United Kingdom; 3 Nottingham Arabidopsis Stock Centre, School of Biosciences, The University of Nottingham, Nottingham, United Kingdom; 4 Centre for Analytical Bioscience, School of Pharmacy, The University of Nottingham, Nottingham, United Kingdom; 5 Princess Nourah Bint Abdulrahman University, Department of Nutrition and food science, College of Home Economics, Riyadh, BOX: 84428 Saudi Arabia; 6 Robinson Research Institute, Medical School, University of Adelaide, Adelaide, Australia; 7 Nutricia Research, Utrecht, The Netherlands; 8 Department of Pediatrics, University Medical Centre Groningen, University of Groningen, Groningen, The Netherlands; 9 VA Endocrinology and Diabetes Division, VA Greater Los Angeles Healthcare System, and Department of Medicine, David Geffen School of Medicine, University of California Los Angeles, California, USA

## Abstract

Brown adipose tissue (BAT) undergoes pronounced changes after birth coincident with the loss of the BAT-specific uncoupling protein (UCP)1 and rapid fat growth. The extent to which this adaptation may vary between anatomical locations remains unknown, or whether the process is sensitive to maternal dietary supplementation. We, therefore, conducted a data mining based study on the major fat depots (i.e. epicardial, perirenal, sternal (which possess UCP1 at 7 days), subcutaneous and omental) (that do not possess UCP1) of young sheep during the first month of life. Initially we determined what effect adding 3% canola oil to the maternal diet has on mitochondrial protein abundance in those depots which possessed UCP1. This demonstrated that maternal dietary supplementation delayed the loss of mitochondrial proteins, with the amount of cytochrome C actually being increased. Using machine learning algorithms followed by weighted gene co-expression network analysis, we demonstrated that each depot could be segregated into a unique and concise set of modules containing co-expressed genes involved in adipose function. Finally using lipidomic analysis following the maternal dietary intervention, we confirmed the perirenal depot to be most responsive. These insights point at new research avenues for examining interventions to modulate fat development in early life.

## Introduction

The association between excessive fat storage (i.e. obesity) and increased risk of metabolic disease, which leads to a reduction in life quality and expectancy, is well documented^1^. To identify modifiable factors that drive unhealthy fat deposition it could be informative to better understand the pronounced developmental changes in adipose tissue that occur soon after birth^2^. This age period is characterised by the rapid activation of nonshivering thermogenesis in brown adipose, an energy-using process involving tissue-specific uncoupling protein (UCP)1^3^, which helps the newborn to achieve a physiological body temperature. Subsequently, brown fat is gradually lost and replaced with white adipose tissue^4^, which contains cells that store energy for later usage as cellular fuel. Over the last decade, high-throughput genome-wide association studies (GWAS), together with gene expression profiling, epigenetic and integrative genomic analysis, have all contributed to a better understanding of adipose tissue biology and its crucial role in the metabolic syndrome^5,6^. Scientific break-through, however, may have been hampered by wrongly assuming that adipose tissue at various anatomical sites is regulated in an identical manner for coping with metabolic challenges during current and later life^2^. To test for site-specific regulation and developmental plasticity of adipose tissue we used sheep, based on the rapid transformation from brown to white adipose tissue characteristics over the first month of life^3^.

The function and development of adipose tissue depots are often studied in isolation^7^, so, potential differences between depots and influence of adjacent organs are not well established^3,6,7^. With recent advances in computer power and functional annotation of transcriptome data, primary genes can be identified based on their pattern of expression across the genome^5,6^. Furthermore, genes with similar co-expression patterns innately cluster together and/or form distinct modules representing pathways involved in the regulation of interdependent biological functions^8^. Differences in the changes in the transcriptome with age between similar tissues may reflect variations in cell type, but also in their function, transcriptional regulation, and responsiveness to external cues^8^. In the present study, we employed a machine learning (ML) algorithm followed by a weighted gene co-expression network analysis in order to find biologically meaningful associations in microarray datasets from the five major fat depots in sheep at 7 (when brown characteristics can dominate) and 28 (when brown fat is scarce) days of age. These measures enabled us to elucidate the distribution of cellular plasticity in response to a nutritional intervention between depots. The maternal diet was modified to cause a shift in the fatty acid (FA) composition of maternal milk achieved through feeding the mothers a supplement of canola oil. The inclusion of canola in dairy ruminants feed has been reported to reduce the omega-6/omega-3 FA ratio and conjugated linoleic acid (CLA) in milk, both which are properties that have been found to up-regulate *UCP* genes in adipose tissue^9^.

## Methods

### Animal Model

All of the procedures were performed with full institutional ethical approval from the University of Nottingham as designated under the United Kingdom Animals (Scientific Procedures) Act, 1986. All laboratory procedures were carried out at The University of Nottingham under the United Kingdom code of laboratory practice (COSHH: SI NO 1657, 1988). For this study, thirteen twin-bearing (non-identical) Bluefaced Leicester cross Swaledale ewes were randomly assigned immediately after giving birth to receive their a standard diet of roughage and concentrate throughout lactation (control, n = 5) or received the same diet supplemented with 3% canola oil (i.e. 45 g in 1500 g of concentrate) (n = 8). All mothers delivered spontaneously at term (~147 d). One (sex-matched) twin from each mother was randomly assigned to be humanely euthanased at 7 days and adipose tissues sampled from the epicardial, perirenal, sternal, subcutaneous and omental depots. Tissues were quickly dissected and weighed before being sectioned and snap frozen in liquid nitrogen for storage at −80 °C. Additional representative sections were fixed in 10% v/v formalin and embedded in paraffin wax for histological analysis. Each remaining twin was reared naturally with their mother until 28 days of age when they were humanely euthanased, and adipose tissue sampled. Samples of the mother’s milk were also taken manually from each mother on days 7 and 28 at ~08.00 h prior to euthanasia of the offspring. The milk was transferred into two sterile 15 ml tubes (Greiner Bio-One, Gloucester, UK) and stored at 80 °C until being shipped on dry ice for analysis of milk FA composition.

### Immunohistochemistry

Tissue sections were prepared as previously published^10^ and stained using haematoxylin and eosin and for UCP1. At least 20 slides per animal alongside a negative control were labelled with a random identifier, loaded into the Leica BondMax IHC slide processor (Leica Microsystem), and run on an automated software program (Vision Biosystems Bond version 3.4A) using bond polymer refine detection reagents (Leica Microsystem) and a 1:500 dilution of primary rabbit polyclonal antibody to UCP1 (ab10983, Abcam).

### Western blotting

The relative abundance of UCP1, voltage dependent anion channel 1 (VDAC) and cytochrome c were determined in the perineal and sternal adipose tissue samples as previously described^11^. This analysis was not performed on epicardial fat as insufficient sample was available. All data was corrected against the density of staining for total protein. Each antibody gave a signal at the correct molecular and the specificity of binding for each anti-body was confirmed using non-immune rabbit serum.

### RNA isolation, quantification, and quality control

For RNA isolation from each fat depot from 5 animals within each nutritional group, and a 100 mg was used from samples collected at 7, and 1000 mg from taken at 28 days of age, respectively. These were mixed with 2 ml of TRI reagent (Sigma-Aldrich). Total RNA was extracted using the RNeasy Plus kit (Qiagen) according to the manufacturer’s instructions and its quantity measured with a NanoDrop ND-1000 Spectrophotometer (Thermo Scientific). Optical density ratios (260/280 nm) were >1.9 for all samples. Total RNA quality was assayed by the Agilent BioAnalyzer RNA 6000 Nano Kit (Agilent Technologies) and only used if distinct ribosomal peaks measured (i.e. RIN > 7).

### Transcriptome profiling with Affymetrix GeneChip

Representative samples of mRNA were labelled and hybridized onto Human Genome U133A plus 2 arrays according to manufacturer’s recommendations using the GeneChip 3′ IVT Express kit (Affymetrix). The Affymetrix Human U133 + 2 gene chip array can be used to study ovine tissues^11^ and detection was performed using a GeneChip Scanner 3000 7G.

### Gene expression array analysis

Normalization and network analyses were undertaken using free and open source packages from the R project (http://cran.r-project.org/) unless otherwise stated. We used the open source Bioconductor community (http://www.bioconductor.org/) with the function “gcrma” embedded in the “affy” package for pre-processing data, including background correction, normalization, and probe match verification. For statistical analysis of gene expression, we used the “limma” library, which enabled us to perform empirical Bayesian statistical modelling between the 5 adipose tissue depots and the effect of the maternal nutritional intervention. For all other statistical analysis, unless otherwise stated, we applied the FDR approach and considered q ≤ 0.05 as significant^12^. The R-package “gplots” was used to assess fold changes, and make heat maps, and expression plots.

### Accession numbers

All original microarray data were deposited in the NCBI’s Gene Expression Omnibus (accession GSE115799).

### Selection of informative genes using the RF-PSOL algorithm

The ML algorithm was prepared using the R package ml-DNA, of which the PSOL algorithm was used to correctly identify ~95–98% of “informative” genes^13^. It employed a “training dataset” comprising the top 100 genes selected through empirical Bayesian statistical modelling between each different tissue at each age. Each result from the classifier or the prediction accuracy of the RF was tested using the 5-fold cross-validation method^14^. The validity for each selection cycle of interactions was assessed by values of the area under the curve (AUC) received from operating characteristic analysis or ROC (i.e. the two-dimensional plot between the false (x axis) versus the true positives rate (y axis) at all possible thresholds). The AUC values ranged from 0 to 1, with a higher AUC indicating better prediction accuracy for the random forest model.

### Gene Co-expression Network Construction

Datasets from both age groups (i.e. 25 samples, comprising 5 different adipose tissues depots from 5 animals) were constructed separately using a standard workflow as recommended by weighted correlation network analysis (WGCNA)^15^. We used a signed weighted correlation network for both age groups and the resulting Pearson correlation matrix was transformed into a matrix of connection strengths (e.g., an adjacency matrix) using a power of 19. For the dynamic tree-cutting algorithms, a merging function was set at 0.25, which identified 11 modules at 7 days and 14 modules at 28 days of age. To better describe molecular outgoing events in each age group dataset, our analysis was restricted to genes selected as informative by the ML-based filtering process^13^.

The function ‘module Preservation’ added into the WGCNA R package provides a reliable module preservation statistics base on the generation of 200 random permutations between two independent datasets. These permutations allow the calculation of a series of network Z-scores for statistical properties such as network connectivity and density. By averaging these Z-scores in a single Z-score or Z-summary, this value can be used as an indication of relationship preservation between genes in two independent networks. For example, a Z-summary for a module that scores >10 could be interpreted as strongly preserved (i.e. no changes in the topology), or scores between 2 and 10 are considered to be moderately preserved, or <2 indicates that the relationship between the genes are not preserved^15^.

### Gene ontogeny analysis

Functional annotation was performed with the WEB-based GEneSeTAnaLysis Toolkit (or WebGestalt) and all genes within each module were analysed. The GO terms with a FDR < 0.05 and enrich >5 genes per classification^16^.

### Determination of milk fat content and composition

Milk fat concentration was determined by methods following Bligh & Dyer^17^. High-resolution capillary gas-liquid chromatography was used to determine the composition of short- to long chain FAs in milk fat as previously described^18^. A total of 46 FAs were analyzed this way and included saturated FAs: C4:0, C6:0, C8:0, C10:0, C11:0, C12:0, C13:0, C13ai, C14:0, C14ai, C15:0, C15ai, C16:0, C16ai, C17:0, C18:0, C18i, C20:0, C20i, C21:0, C22:0, C23:0, monounsaturated FAs: C14:1n-5, C15:1n-5, C16:1n-7, polysaturated FAs, omega (n)-3: C18:3n3, C18:4n-3, C20:3n3, C20:5n-3, C22:5n3, C22:6n3. Omega (n)-6: C18:2n6tr, C18:2n6, C18:3n-6, C20:2n-6 C20:3n6, C20:4n6, C22:2n-6, C22:4n-6, C22:5n-6 and omega (n)-9: C16:1n-9/7t, C18:1n-9, C20:1n-9, C22:1n-9, C24:1n-9. Results are expressed as % milk fat. All data were evaluated using the “Limma” and “gplots” in a similar fashion as described above for with the microarray analysis.

### Determination of adipose tissue lipid composition

Adipose tissue samples (100–200 mg) were ground in Retsch ball mill for 3 min using 6 mm stainless steel balls, all pre-chilled to −18 °C. Ice cold chloroform: methanol (2:1) was added (0.5 mL) and the slurry was agitated at room temperature for 20 min, and centrifuged for 10 min at 10,000 × g at 4 °C. The lower layer was removed and dried in a centrifugal evaporator. The samples were reconstituted in 100 µL chloroform:methanol 1:2 centrifuged, then decanted into amber glass LC vials with inserts and stored at −80 °C until LC-MS analysis. Lipidomic QC samples were created from pooling equal volumes of all sample extracts. The extracted lipids were injected (5 µL) onto an Agilent Poroshell 120 SB-C18 50 × 2.1 mm (2.7 µm particle size) with guard, held at 45 °C, and eluted using 0.1% aqueous ammonium acetate (A), to 0.1% aqueous ammonium acetate /acetonitrile/isopropanol gradient (1:1:8) (B) using gradient elution. A ThermoScientific Accela modular HPLC system (Hemel Hempstead, UK) was used at a flow rate of 0.45 mL/min. Ions in the range *m/z* 100 to 1900 were detected using an Exactive series mass spectrometer (ThermoScientific Hemel Hempstead, UK) in electrospray mode with +/−ve switching at a resolution setting of 25000. Data was normalised to the total ion count, pre-processed and exported to Excel for further processing using Progenesis QI software (Progenesis, Newcastle on Tyne, UK). A product ion prediction tool from Lipidmaps (Lipid MS Predict) was use to assist spectral interpretation. The identities of selected isobaric lipid species were subsequently elucidated by generating MS/MS spectra (nominal mass) using the same LC method with a Thermo Scientific LTQ Velos ion trap mass spectrometer using equivalent electrospray source settings and with a collision energy of 40^19^.

## Results

### Changes in the cellular landscape of brown and white adipose tissue during the first four weeks of postnatal life

**Figure 1:**
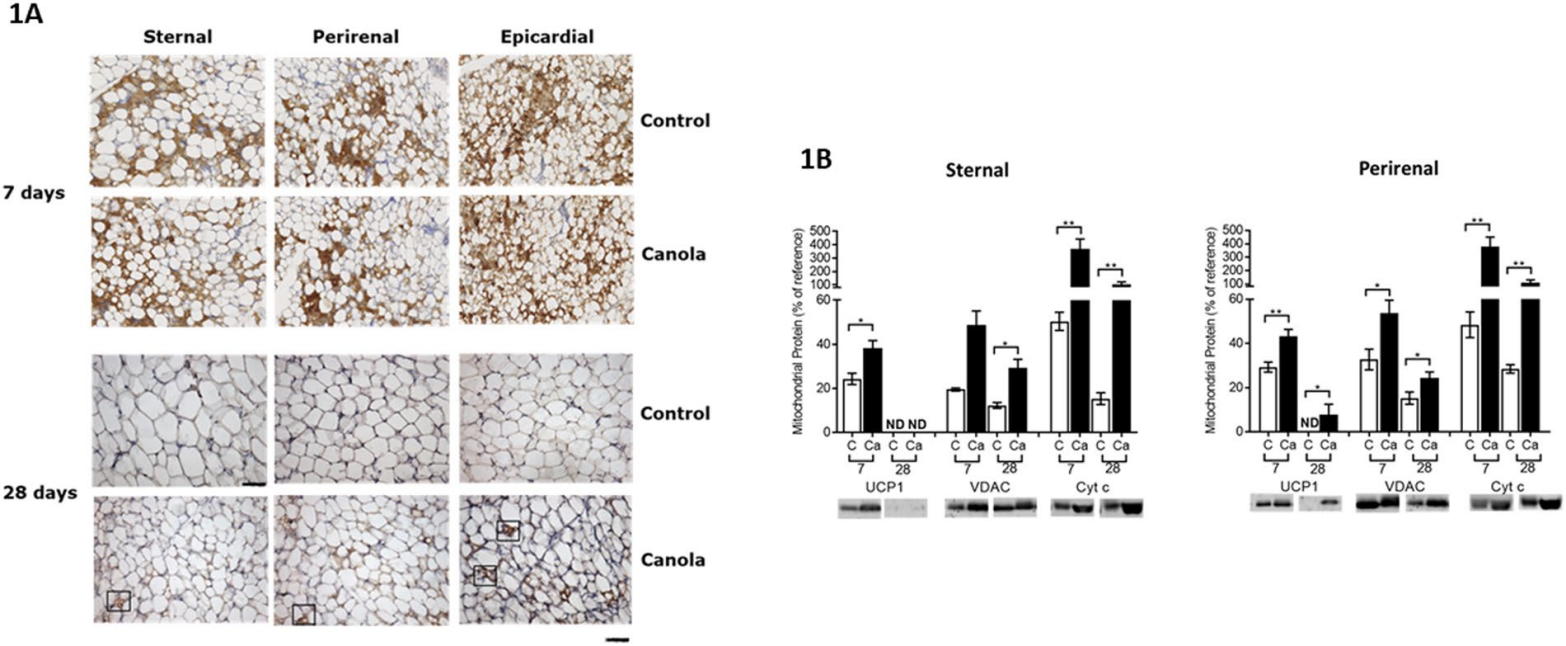
(**A**) Representative immunohistochemical detection of uncoupling protein (UCP)1 from sternal, perirenal and epicardial, sampled from 7 and 28 day old sheep. Rectangular outlines indicate clusters of uncoupling protein 1 (UCP) positive cells found in the epicardial adipose tissue at 28 days (scale bar = 50 μm; Magnification 40x) and (**B**) mean mitochondrial protein abundance as determined by western blotting in adipose tissue sampled from 7 and 28 day old offspring born to mothers fed a control diet (n = 5) or supplemented with 3% canola oil (n = 8). Values are means with their standard errors significant differences between dietary groups at the same age denoted by *p < 0.05; **p < 0.01. UCP, Voltage dependent anion channel 1, VDAC.

To establish which fat depots could be classified as being brown and whether the rate of loss of brown adipocytes occurs at similar rates in those depots, we first examined the distribution of UCP1 by immunohistochemistry. At 7 days of age, UCP1 was more abundant in perirenal and sternal than in epicardial adipose tissue (Fig. 1A) and was not present in subcutaneous or omental fat (not shown). UCP1 abundance was substantially reduced by 28 days of age, although more UCP1 signal remained in the epicardial compared to other depots. In addition, the rate of loss of UCP1 appeared to be delayed by the addition CLA to the mother’s diet. We then further determined whether this response was specific to UCP1 using western blotting, in those brown fat depots of which we had sufficient tissue i.e. perirenal and sternal (Fig. 1B). This demonstrated that the rate of loss of both UCP1 and VDAC were delayed in both depots at 7 and 28 days of age (Fig. 1B), whilst the amount of cytochrome C was enhanced.

**Figure 2:**
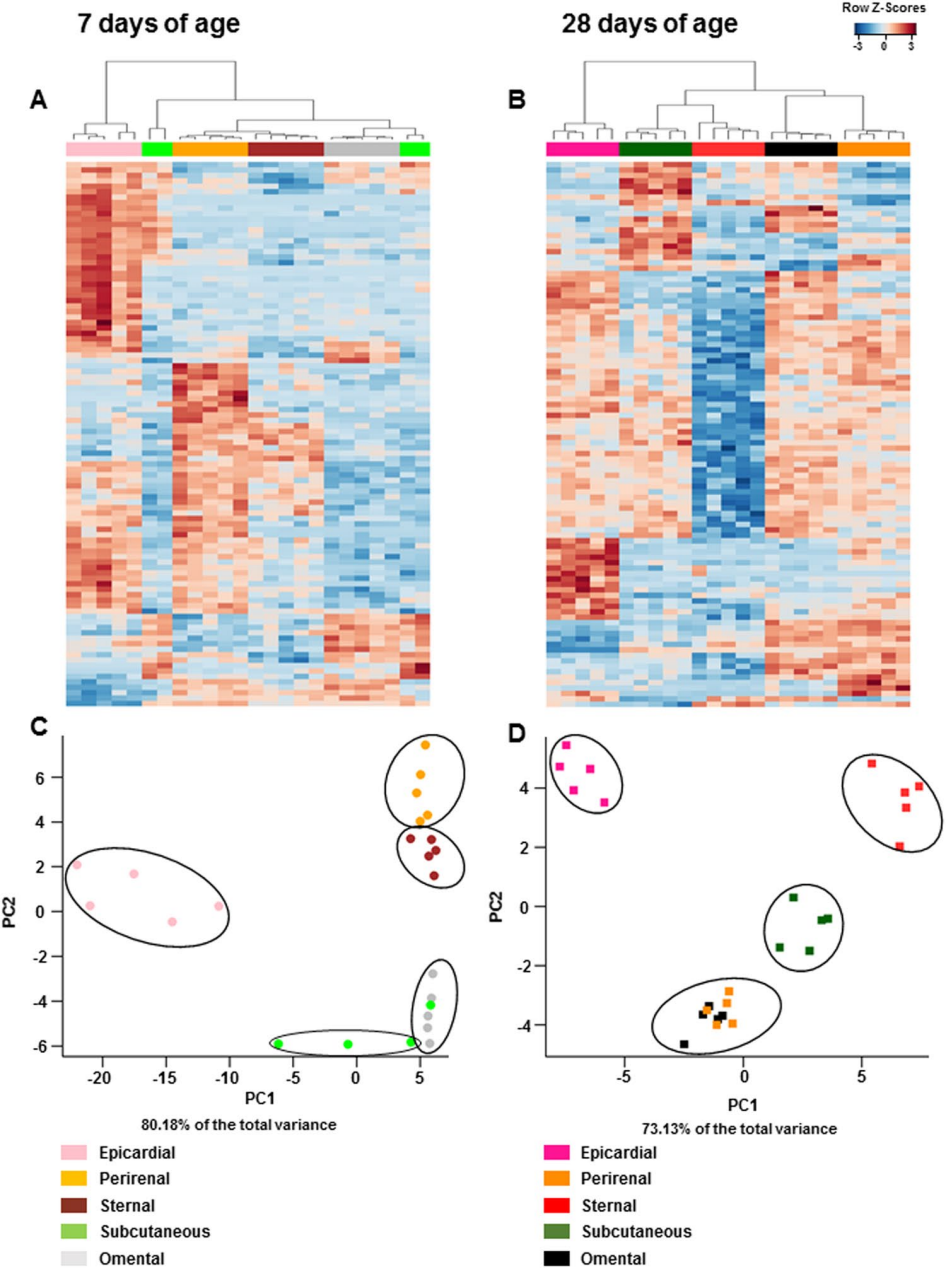
Comparison in gene expression of five adipose tissue depots at (**A**) 7 and (**B**) 28 days of age. Heat map and unsupervised hierarchical clustering dendrograms are shown for the top 100 differentially-expressed gene transcript comparisons identified by microarray analysis (average linkage, Euclidean distance metric) as selected by eBayes moderated t-statistics (FDR < 0.05). Gene expression was transformed to a Z-score, and blue represents a relative is a decrease and red an increase in gene expression between each depot at the same age. Principal component analysis (PCA) of gene expression data from five different adipose tissue depots from each animal was performed using 10055 data sets that passed the variance test QC at (**C**) 7 and (**D**) 28 days of age. Each sample is represented by a sphere (7 days) or rectangle (28 days) and color-coded to indicate the age and tissue to which it belongs.

In order to identify changes in gene regulation with age, we performed a transcriptomic comparison between all 5 depots. At 7 days of age, 839 genes were significantly upregulated between depots (false discovery rate (FDR) ≤ 0.05; Supplement: Dataset 1) and the greatest difference was between epicardial and omental fat (429 significant genes, FDR ≤ 0.05; Fig. 2A). Only perirenal adipose tissue, however, exhibited a consistent upregulation of *UCP1* and other thermogenesis-related genes compared with the omental and subcutaneous adipose depots, which were populated primarily by white adipocytes (Supplement: Dataset 1). These results indicate local differences in transcriptional regulation, which is indicative of substantial changes in cell population between fat depots during postnatal life. This is in accord with their divergent developmental ontogeny between depots. In this regard epicardial, perirenal and sternal are all present in the fetus^20^, whereas omental and to a lesser extent subcutaneous fat only appears after birth^21^.

Next, we performed a clustering analysis of the transcriptomic data at 7 days of age. Unsupervised hierarchical clustering and principal component analysis (PCA) revealed that each depot forming a distinct cluster (Fig. 2), with the first two components explaining 80.2% of the total variance in the data set. This suggests a strong fit between the computational model and the existence of intrinsic differences in gene expression between depots. PCA cluster analysis further showed that the pro-thermogenic perirenal and sternal fat depots clustered together as did subcutaneous and omental depots (white fat depots) whereas epicardial adipose tissue was separate from other depots (Fig. 2C).

We repeated these statistical analyses at 28 days of age to identify the primary changes in gene expression, and identified 2059 differentially expressed genes between depots (FDR ≤ 0.05; Supplement: Dataset 2). At this time point, sternal adipose tissue showed a more pronounced downregulation of the transcriptional architecture compared with the other fat depots (Fig. 2B). Similar to our observation at 7 days of age, each depot had a distinctive pattern of gene expression. Genes within the perirenal depot clustered together with those from omental fat and close to those from the subcutaneous depot, but away from the epicardial and sternal depots. Overall these samples formed three stable clusters and the first two components of the PCA analysis accounted for 73.1% of the variance observed in gene expression between depots (Fig. 2D). In summary, clustering analyses demonstrated that anatomical location determines transcriptome differences which are modified with age. The sternal and perirenal depots exhibited the greatest transcriptomic remodeling, reflecting changes in their biological/metabolic functions with age^4^.

### Use of ML and gene network analyses for identifying the transcriptional architecture changes between adipose tissue depots with age

To determine the magnitude of potential changes in gene function between depots during early life, we combined computer-assisted learning algorithms with weighted gene co-expression network analysis. Through a reiterative process of error minimisation and supervised learning algorithms (i.e. random forest (RF)), the optimal gene expression pattern for each adipose depot was established^14^. Tissue gene expression datasets were submitted for each age group, the RF approach enabled identification beyond a 98% certainty of the informative genes with an overall accuracy of 95–100%.

**Figure 3:**
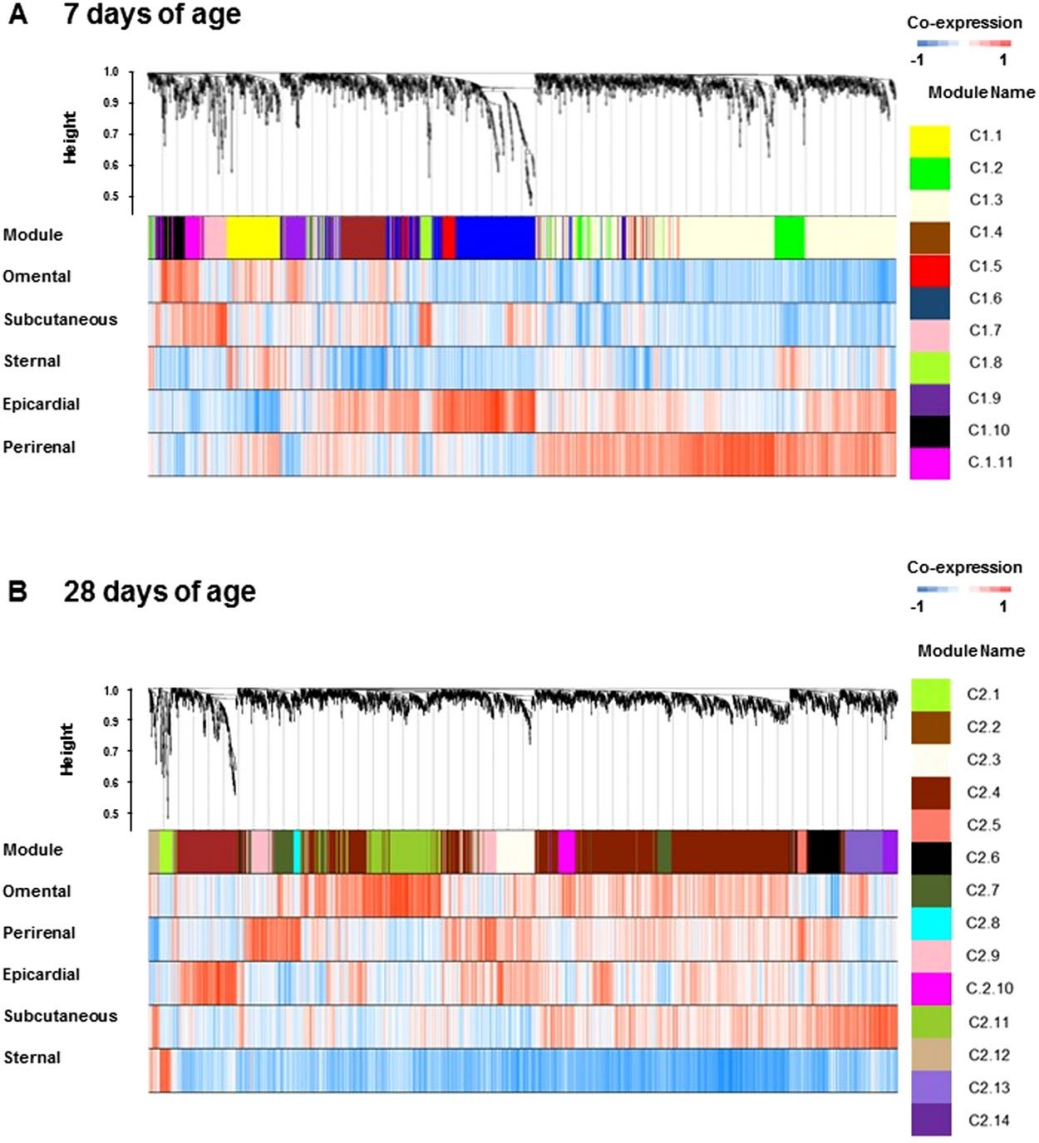
Co-expression dendrogram analysis from the five adipose tissue depots sampled at either (**A**) 7 or (**B**) 28 days of age. In each dendrogram, the first row is subdivided into co-expressed modules founded in each age group. Rows 2 to 6 show the differential expression relationships between module genes and the adipose depot. The relationship of each gene with the assigned module is colour coded from blue (negative co-expression) to red (positive co-expression).

The number of informative genes varied with age indicating different response patterns of the transcriptomes between depots, with 2274 and 3339 genes identified as informative at 7 and 28 days of age, respectively. Next, we used both data sets of informative genes to generate two independent weighted co-expressed networks for each age. We identified 11 distinct modules (designated as C1.1–11; Fig. 3A and Supplement: Dataset 3) at 7 days of age and 14 modules of co-expressed genes at 28 days (designated as C2.1–14; Fig. 3B and Supplement: Dataset 4). Further analyses demonstrated that most gene modules corresponded to different transcriptional or metabolic functions performed in each depot, whereas the intensity of these events varied with age.

### Functional enrichment of gene clusters obtained at 7 days of age

**Figure 4:**
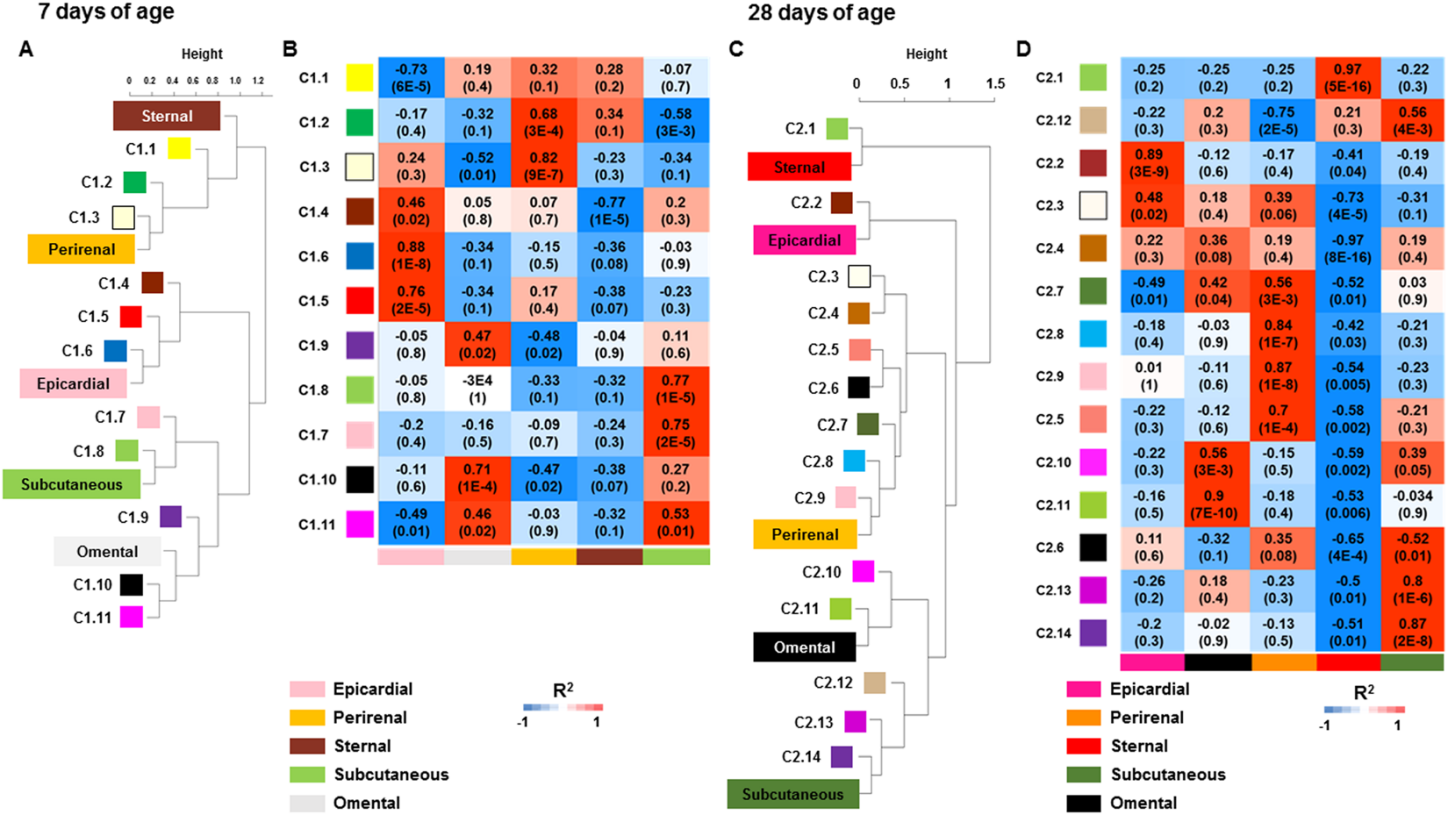
Summary of adipose tissue depot- and age-specific functional organisation of modules within each gene network. They were related individually by their first principal component, referred to as the module eigengene (ME). Each dendrogram illustrates the modules of co-expressed genes and their positive alignment within the ME at (**A**) 7 and (**C**) 28 days of age. The height (X-axis) indicates the magnitude of correlation expressed as Euclidean distances. Heat maps represent the correlation (and corresponding p-values) between co-expressed modules for each fat depot at (**B**) 7 and (**D**) 28 days of age. The colour scheme, from blue to red, indicates the magnitude of correlation, from low to high. Regional-specific modules identified as being highly correlated (i.e. over-expressed) for each adipose depot are shown in the columns.

The gene network analyses demonstrated that the sternal and perirenal depots shared common transcriptional functionalities, although they were more pronounced in perirenal fat (Fig. 4A and B). Many of these modules (i.e. C1.1, C1.2 and C1.3) contained genes involved in regulating mitochondrial biogenesis and aerobic respiration. The C1.3 and C1.2 modules showed several gene ontology enriched terms related to oxidation-reduction processes and mechanisms of mitochondrial regulation of mRNA translation, respectively (Supplement: Dataset 5). However, the separation of both co-expressed modules suggests the potential existence of multiple transcriptional loops regulating mitochondrial genes^5,22^. These transcriptional separations could potentially allow brown adipocytes to regulate the expression of mitochondrial genes independent of UCP1 expression^23^.

**Figure 5:**
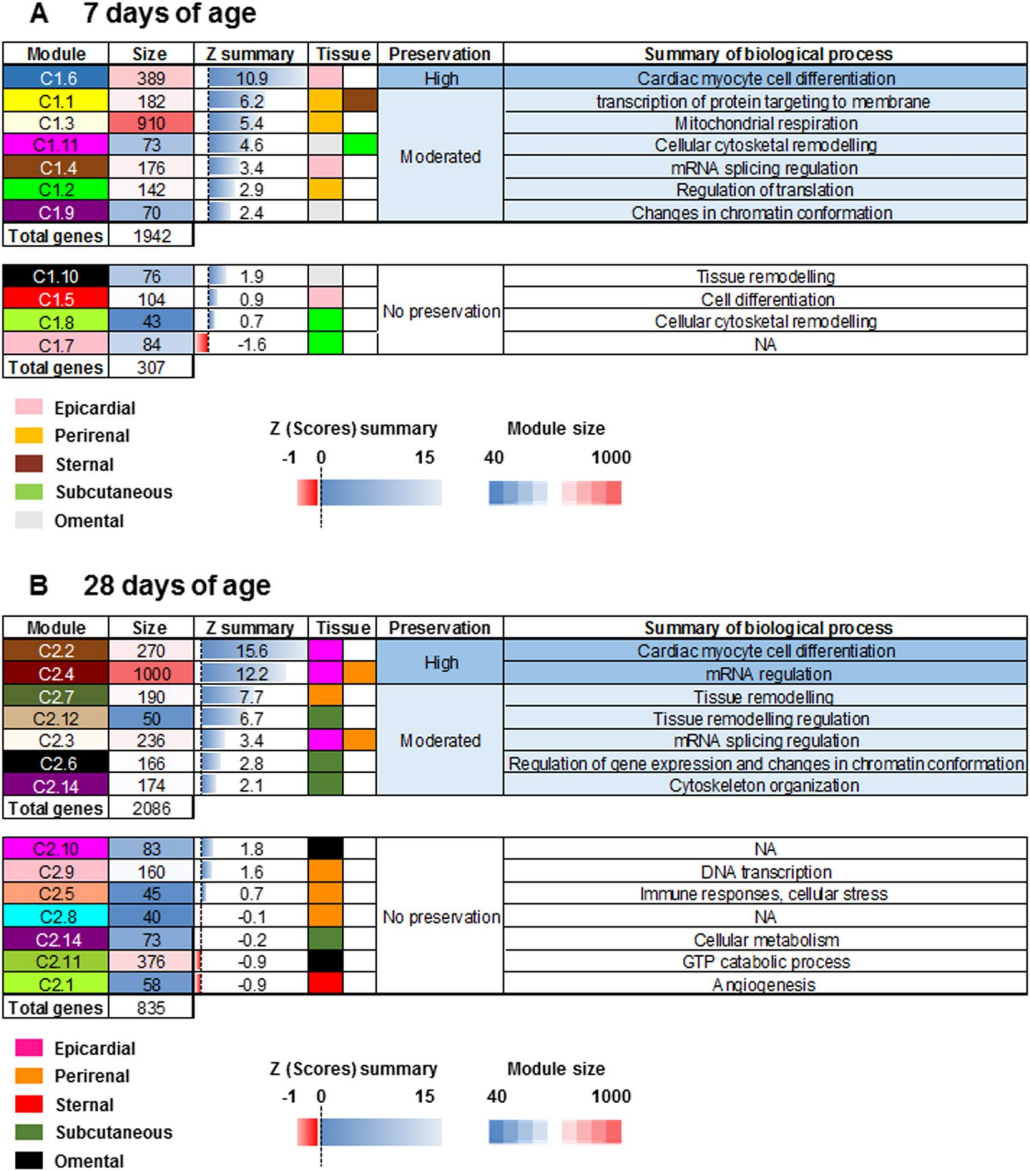
Summary of cross-adipose tissue depot module preservation with age. The test uses a Z score summary of different network properties to determine gene connectivity at (**A**) 7 and (**B**) 28 days of age. Each row represents a module and each column a unique feature of each module including positive alignment with each ME and the number of genes per module. A Z summary value >2 represents a moderately preserved module, and a value >10 provides strong evidence of module preservation.

The majority of gene modules aligning within epicardial adipose tissue coincided with specific stages of changes in transcriptional cardiomyocyte cell differentiation (Fig. 5A)^24^. For example, the C1.4 module showed hallmarks of mRNA and DNA processes associated with the maintenance of stem cell pluripotency^24^. Module C1.6 had a large transcriptional signature usually found in mature cardiomyocytes, suggesting that these transcripts reflect an advanced stage in cell differentiation^25^. All these findings are in agreement with the known pluripotency of adipose tissue-derived stem cells towards differentiation into cardiac myocyte-like cells or brown adipocytes^26^.

Modules, C1.7 and C1.8, both describe transcription events occurring mainly within subcutaneous adipose tissue (Fig. 4A), and contain genes functionally linked to myocyte precursors^26^. These are considered to give rise to brown adipocytes and a subset of white adipocytes populating subcutaneous fat (Supplement: Dataset 5). Finally, we identified modules C1.9, C1.10 and C1.11 that were enriched within omental fat and contained genes linked to carbohydrate metabolism, cytoskeleton composition and tissue remodeling mediated through activation of the immune system (Supplement: Dataset 5)^27^.

### Changes in transcriptional control and its consequences for adipocyte function with age

To address whether the differences in transcriptome regulation identified between depots at 7 days of age had any phenotypical consequences, we generated a second gene co-expression network at 28 days of age. As observed from the PCA analysis, the perirenal depot underwent the greatest shift in gene expression. Four co-expression modules with selective enrichment in that depot were detected, of which the C2.7 module was also enriched in omental fat (Fig. 4C,D). This common module indicates a major metabolic change as perirenal adipose tissue adapts from a heat production depot populated by brown adipocytes to a white fat depot, storing energy as lipid^4,22^. This module contained many genes associated with FA metabolism, including *GHR*, *ABHD5*, *DECR1* and *PPARG* that also regulates adipocyte differentiation (see Supplement: Datasets 4 and 6)^28^. In addition, this module revealed that genes associated with white adipose tissue expansion were co-expressed, including angiogenic genes (*HOXA5*, *HIF1A*) and pre-adipocyte precursor genes (*HOXB6*, *HOXB8*, *HOXB5*; Supplement: Datasets 4 and 6)^29,30^. Other genes enriched in perirenal fat were those in module C2.5, and included transcripts associated with activation of inflammatory responses and endoplasmic reticulum, indicating cellular stress (Supplement: Dataset 6)^27^. The last two modules aligned within this depot, C2.9 and C2.8, had large signatures associated with cell division and mRNA transcriptional regulation, suggesting an increase in cell diversification or differentiation (Fig. 4C and Supplement: Dataset 6)^3,31,32^. Conversely, in sternal fat, these cellular events appeared to be repressed (Supplement: Dataset 2), and the only cluster positively aligned was C2.1, which was enriched with genes indicating chemical or hormonal responsiveness and those leading to tissue angiogenesis (Fig. 5B and Supplement: Dataset 6)^30,33^. As observed at 7 days, the epicardial adipose tissue also exhibited modules enriched with genes with a large genetic signature associated with cardiomyocyte cell differentiation such as module C2.2 (Fig. 5B)^26^.

Modules C2.10 and C2.11 showed significant relationships in omental adipose tissue (Fig. 4C,D)^32^, and exhibited over-representation of transcripts linked to the balance between anabolic and catabolic pathways occurring in mature adipocytes (Supplement: Dataset 6)^34^. Finally, in subcutaneous adipose tissue, most genes were assigned to modules C2.12, C2.13 and C2.14 and these were associated with cell differentiation, growth and remodelling^30^.

### Changes in gene networks represent functional adaptations of different adipose depots during development

To explore the biological relevance of each module in more detail, we applied a statistical approach based on a series of random permutations between datasets. This enabled us to find evidence of similarities and differences in the network topology at both ages. We found that the majority of genetic interactions persisted at both ages (Fig. 5A,B). At 7 days, 86.4% of all the genes were allocated to a conserved module, including module C1.2 which was enriched with mitochondrial genes such *UCP1* (Fig. 5A). Gene ontology analysis of the four non-conserved modules showed that they all shared functional similarities associated with early stages of cell differentiation and adipose tissue remodelling^30^. At 28 days, 71.4% of all the genes were allocated to a conserved module within the network. The gene ontogeny enrichment analysis from these conserved modules revealed metabolic and transcriptomic processes associated with mature adipocyte function which mainly involve lipid metabolism, immune responses and tissue remodeling (Fig. 5B).

### Functional assessment of changes in transcriptional architecture

In order to evaluate the potential impact of the transcriptional differences between adipose depots, we performed lipidomic and gene expression analyses on perirenal and sternal fat at 28 days of age in offspring of mothers who consumed a diet supplemented with 3% canola oil. By this point of lactation milk from supplemented mothers had developed an altered FA profile without changes in the total fat content. Besides, the maternal nutritional supplementation had no effect on growth or body weight and of the offspring. Features of the FA profile that had changed in relation to the control group according to predictions included the statistically significantly lowered proportion of linoleic acid and a lowered omega-6/omega-3 FA ratio (Supplement: Dataset 7 and Supplement: Fig. 1). The supplemented milk exhibited similar omega 3 FAs but a lower arachidonic (C20:4n6) and γ-linolenic FA (C18:3n6) content. Dietary supplementation produced a localised change in the lipidome of perirenal fat (Supplement: Dataset 8 and Supplement: Fig. 2). Overall, the intervention led to differences in the relative abundance of 28 day perirenal adipose tissue spectrometry lipid masses. Amongst those that we were able to identify, we observed decreased proportions of phosphatidylcholines (PC; a major constituent of cell membranes) and long chain FAs (carbon chain length >20). In contrast, in sternal adipose tissue, there were no differences in lipid composition between the intervention and control groups at 28 days of age. Overall, microarray analyses of both fat depots revealed that only the genome of perirenal fat responded to the nutritional intervention. Data mining (Supplement: Dataset 9) results thus showed a significant increase in the expression of only 4 genes. Interestingly, when comparing the most differentially regulated genes, effects of the intervention on the down regulation of *NR3C1* were highlighted. This gene encodes for the glucocorticoid receptor and is directly associated with inflammatory responses, cellular proliferation, lipid metabolism, cell differentiation and more importantly the modulation of thermogenesis in brown fat^35,36^. Potentially, due to the relatively short duration of intervention and/or by modest amount of canola oil added to the diet, we could not observe substantial changes in UCP1. Taken together, these results demonstrate that the cellular architecture of perirenal fat is unique among fat depots in that it can undergo pronounced remodeling and is sensitive even to a modest nutritional stimulus.

## Discussion

We have shown profound differences in gene expression profiles between the major fat depots in sheep through early postnatal life, coincident with the transition from brown to white fat depots^3^. The developmental changes markedly differed between depots despite them showing a similar macroscopic morphology at each developmental age. For example, at 28 days when in sheep fat is considered primarily white^3^, each adipose depot kept a distinct expression profile. This appeared to determine its capacity to respond to modification of the composition of the mother’s milk. Adipose tissue has been considered a metabolic organ with important functions beyond lipid storage but the extent to which this varies between depots especially during development is largely unexplored. Our new data support the concept that adipose tissue functions not as one metabolic organ, but as several autonomic organs which appear to have distinct functions^32^.

By using a computer-assisted supervised learning algorithm, we demonstrate that during postnatal development each fat depot contains a transcriptome which forms dynamic networks with unique sets of genes^8^. Over time these gene networks can undergo profound reorganisation by accommodating novel members and/or losing some of their original components^8^. Whilst ontogenic plasticity can be driven entirely by intrinsic factors, survival value in fluctuating environments can be enhanced by responsiveness to extrinsic factors^3^. However, both types of plasticity are prone to error and maladaptation which can ultimately lead to obesity, increasingly threatening metabolic health^1^. Understanding how environmental factors, particularly during early life, interfere with pathways of energy utilisation or storage is one of the most important intermediate goals in obesity prevention. By examining the postnatal development of adipose tissue through gene network analysis, we have been able to construct novel biological interpretations^8^ specific to each fat depot over the period when any large mammal needs to respond to environmental, nutritional and physiological challenges^3^. We, therefore, explored dynamic changes in gene regulation and identified the main regulatory relationships. These have crucial regulatory roles so each separate adipose tissue depot can differentiate and adapt, potentially enabling the different genes involved to modulate metabolic homeostasis^32^. Despite recent efforts to elucidate the cellular and transcriptome composition of different fat depots^4,23^, the influence of genetic, endocrine or environmental factors on fat development remains largely unknown. However, in our study we observed that the co-expression modules within networks show a depot-specific pattern enriched with genes performing specific functions.

Studies on adipose tissue function during early postnatal life have mostly focused on explaining the loss of genes associated with cellular thermogenesis, especially *UCP1*^3^. Our comparison between the three depots enriched with brown adipocytes suggests the existence of different networks in the regulation of mitochondrial activity^5^. The expression of mRNA is regulated by a balance between transcription and mRNA degradation, and the C1.2 module captures this complexity in the control of UCP1^37^. We found transcription factors that stimulate a cell’s transition from myoblastic precursors to brown fat cells, including C/EBPβ and EP300^22,38^. Other members of this module were EIF4B, EIF4G3, EIF3D and EIF3G, known transcriptional factors that downregulate mRNA transcription of UCP1 in response to raised temperature^39^. We also found a large number of ribosomal proteins co-expressed with UCP1 that are similarly regulated, including RPS5 and RPS9. These comprise part of the original mitochondrial protein assembly machinery^40^. UCP1 is also regulated by AU-rich elements, which are mRNA binding proteins^37^. In humans, it has been estimated that less than 8% of genes are regulated in this manner, raising the possibility that mRNA binding proteins such as ZFP36L1 and DHX32 co-expressed with UCP1 could potentially degrade this gene^37,41^. ZFP36L1 specifically binds at its 3′-UTR mRNA site and recruits the Cnot7-Tob-BRF1 axis, resulting in mRNA destabilisation^41^. These genes are co-regulated with multiple transcripts involved in energy metabolism and mRNA transcription allocated to the C1.1 and C1.3 modules, which are both related to the functional changes observed in perirenal fat up to 28 days of age.

Another novel finding is the relationship between epicardial adipose tissue and genes associated with cardiomyocyte cell differentiation^24^. Cardiomyocytes rapidly differentiate and proliferate during fetal life, but exit the cell cycle soon after birth, limiting the ability of the heart to restore function after any significant injury^26^. There are reservoirs of multipotent stem cells in most fat depots which can differentiate *in vitro* into brown adipocytes and also, as observed, into cardiomyocytes^25^. As shown in Fig. 5, the highest biologically expressed genes were those of cardiac myocytes in epicardial fat cells in module C1.6 at 7 and module C2.2 at 28 days, respectively. One pathway to brown adipocyte differentiation is through chronic adrenergic stress, as observed in rodents after prolonged cold exposure^39^ and in patients with severe skin burn injuries^42^. We have previously observed that epicardial fat maintains a significant number of brown adipocytes, in children with congenital heart disease^43^. It is, therefore, possible that the fate of these multipotent stem cells, or their ability to differentiate into other cell types, could be regulated by specific growth factors supporting normal physiological development or enabling them to respond to disease^24,43^.

Our study confirms that the perirenal and sternal depots follow different development patterns^20^. Although *UCP1* expression ceases in both depots with age, only perirenal fat exhibits the major hallmarks of white adipose tissue development^7^. This insight was further substantiated by the observation that, when offspring consumed milk from mothers that had received a 3% supplement of canola oil, only the cellular lipidome of the perirenal adipose tissue was modified. This was accompanied with the retention of UCP1 up to 28 days of age, that is likely to be mediated by differences in milk lipid profiles found between control and intervention groups. Supplementation of canola oil in the maternal diet did not have a direct effect on omega 3, but limited the accretion of two omega 6 FAs, γ-linolenic acid (C18:3n6) and more significantly of arachidonic acid (C20:4n6). This essential FA is metabolized by a transcellular process using cyclooxygenases to induce prostaglandin synthesis, thus triggering a pro-inflammatory response^44^. The changes in arachidonic acid in perirenal adipose tissue may explain in part the differences in membrane architecture and the up-regulation of genes related to inflammation between groups. Downstream, we observed that these changes in FA milk profiles were accompanied with reduced NR3C1 gene expression in perirenal fat. This is the transcript of the type 2 glucocorticoid receptor mRNA, which is important in regulating the actions of glucocorticoids in most tissues^3^. Furthermore, glucocorticoids are not only necessary for adipocyte differentiation they also modulate thermogenesis in a species and depot specific manner^35^. Taken together, our observations suggest that differences in the tissue microenvironment, possibly dictated by nearby endocrine organs such as the adrenals, determine changes in the metabolic/phenotypic characteristics of existing fat cells^6,32^.

In conclusion, adipose tissue depots differ dramatically in terms of their gene expression signature, differentiation ability, cellular composition, and capacity to respond to local environmental stimuli^45^. Perirenal adipose tissue shows the greatest propensity to differentiate and respond to an external stimulus. In contrast, fat depots such as sternal and epicardial do not exhibit an adipogenic profile and would therefore, complete their normal programmed development. The data presented here suggest that microarray gene expression in combination with advanced data analytic tools provide a robust and accurate approach for producing adipose depot-specific gene signatures. Moreover, this approach could enhance our ability to identify and manipulate specific characteristics of adipose tissue in different anatomical locations.

## Electronic supplementary material

Supplementary Figures and Information

Dataset 1

Dataset 2

Dataset 3

Dataset 4

Dataset 5

Dataset 6

Dataset 7

Dataset 8

Dataset 9

## References

[1] Gregg EW, Shaw JE (2017). Global Health Effects of Overweight and Obesity. N Engl J Med.

[2] Spalding KL (2008). Dynamics of fat cell turnover in humans. Nature.

[3] Symonds ME, Pope M, Budge H (2015). The Ontogeny of Brown Adipose Tissue. Annu Rev Nutr.

[4] Rockstroh D (2015). Direct evidence of brown adipocytes in different fat depots in children. Plos One.

[5] Claussnitzer M (2015). FTO Obesity Variant Circuitry and Adipocyte Browning in Humans. N Engl J Med.

[6] Lotta LA (2017). Integrative genomic analysis implicates limited peripheral adipose storage capacity in the pathogenesis of human insulin resistance. Nat Genet.

[7] Gesta S, Tseng YH, Kahn CR (2007). Developmental origin of fat: tracking obesity to its source. Cell.

[8] Barabasi AL, Oltvai ZN (2004). Network biology: understanding the cell’s functional organization. Nat Rev Genet.

[9] Welter KC (2016). Canola Oil in Lactating Dairy Cow Diets Reduces Milk Saturated Fatty Acids and Improves Its Omega-3 and Oleic Fatty Acid Content. Plos One.

[10] Pope M, Budge H, Symonds ME (2014). The developmental transition of ovine adipose tissue through early life. Acta Physiol (Oxf).

[11] Mostyn A (2003). Ontogeny and nutritional manipulation of mitochondrial protein abundance in adipose tissue and the lungs of postnatal sheep. Br J Nutr.

[12] Wettenhall JM, Smyth GK (2004). limmaGUI: a graphical user interface for linear modeling of microarray data. Bioinformatics.

[13] Ma C, Xin M, Feldmann KA, Wang X (2014). Machine learning-based differential network analysis: a study of stress-responsive transcriptomes in Arabidopsis. Plant Cell.

[14] Breiman L (2001). Random Forests. Machine Learning.

[15] Oldham MC, Horvath S, Geschwind DH (2006). Conservation and evolution of gene coexpression networks in human and chimpanzee brains. Proc Natl Acad Sci USA.

[16] Wang J, Duncan D, Shi Z, Zhang B (2013). WEB-based GEne SeT AnaLysis Toolkit (WebGestalt): update 2013. Nucleic Acids Res.

[17] Bligh EG, Dyer WJ (1959). A rapid method of total lipid extraction and purification. Can J Biochem Physiol.

[18] Kovacs A, Funke S, Marosvolgyi T, Burus I, Decsi T (2005). Fatty acids in early human milk after preterm and full-term delivery. J Pediatr Gastroenterol Nutr.

[19] Ravipati S, Baldwin DR, Barr HL, Fogarty AW, Barrett DA (2015). Plasma lipid biomarker signatures in squamous carcinoma and adenocarcinoma lung cancer patients. Metabolomics.

[20] Henry BA (2017). Ontogeny and Thermogenic Role for Sternal Fat in Female Sheep. Endocrinology.

[21] Saroha V (2017). Tissue cell stress response to obesity and its interaction with late gestation diet. Reprod Fertil Dev.

[22] Kajimura S (2009). Initiation of myoblast to brown fat switch by a PRDM16-C/EBP-beta transcriptional complex. Nature.

[23] Lidell ME (2013). Evidence for two types of brown adipose tissue in humans. Nat Med.

[24] Liu Q (2014). Epicardium-to-fat transition in injured heart. Cell Res.

[25] Boheler KR (2002). Differentiation of pluripotent embryonic stem cells into cardiomyocytes. Circ Res.

[26] Yamada Y, Wang XD, Yokoyama S, Fukuda N, Takakura N (2006). Cardiac progenitor cells in brown adipose tissue repaired damaged myocardium. Biochem Biophys Res Commun.

[27] Lu C, Kumar PA, Fan Y, Sperling MA, Menon RK (2010). A novel effect of growth hormone on macrophage modulates macrophage-dependent adipocyte differentiation. Endocrinology.

[28] Rajakumari S (2013). EBF2 determines and maintains brown adipocyte identity. Cell Metab.

[29] Cantile M, Procino A, D’Armiento M, Cindolo L, Cillo C (2003). HOX gene network is involved in the transcriptional regulation of *in vivo* human adipogenesis. J Cell Physiol.

[30] Park YK (2013). Hypoxia-inducible factor-2alpha-dependent hypoxic induction of Wnt10b expression in adipogenic cells. J Biol Chem.

[31] Lee YH, Petkova AP, Granneman JG (2013). Identification of an adipogenic niche for adipose tissue remodeling and restoration. Cell Metab.

[32] Macotela Y (2012). Intrinsic differences in adipocyte precursor cells from different white fat depots. Diabetes.

[33] Sharkey D (2009). Impact of early onset obesity and hypertension on the unfolded protein response in renal tissues of juvenile sheep. Hypertension.

[34] Pietilainen KH (2011). Association of lipidome remodeling in the adipocyte membrane with acquired obesity in humans. Plos Biol.

[35] Lu NZ (2006). International Union of Pharmacology. LXV. The pharmacology and classification of the nuclear receptor superfamily: Glucocorticoid, mineralocorticoid, progesterone, and androgen receptors. Pharmacol Rev.

[36] Ramage LE (2016). Glucocorticoids Acutely Increase Brown Adipose Tissue Activity in Humans, Revealing Species-Specific Differences in UCP-1 Regulation. Cell Metab.

[37] Takahashi A (2015). Post-transcriptional Stabilization of Ucp1 mRNA Protects Mice from Diet-Induced Obesity. Cell Rep.

[38] Vargas D (2016). Regulation of human subcutaneous adipocyte differentiation by EID1. J Mol Endocrinol.

[39] van Breukelen F, Sonenberg N, Martin SL (2004). Seasonal and state-dependent changes of eIF4E and 4E-BP1 during mammalian hibernation: implications for the control of translation during torpor. Am J Physiol Regul Integr Comp Physiol.

[40] Maier UG (2013). Massively convergent evolution for ribosomal protein gene content in plastid and mitochondrial genomes. Genome Biol Evol.

[41] Adachi S (2014). ZFP36L1 and ZFP36L2 control LDLR mRNA stability via the ERK-RSK pathway. Nucleic Acids Res.

[42] Sidossis LS (2015). Browning of Subcutaneous White Adipose Tissue in Humans after Severe Adrenergic Stress. Cell Metab.

[43] Ojha S (2016). Gene pathway development in human epicardial adipose tissue during early life. JCI Insight.

[44] Smith WL, Song I (2002). The enzymology of prostaglandin endoperoxide H synthases-1 and -2. Prostaglandins Other Lipid Mediat.

[45] Lee YH, Kim SN, Kwon HJ, Granneman JG (2017). Metabolic heterogeneity of activated beige/brite adipocytes in inguinal adipose tissue. Sci Rep.

