# Supplementary material for: Transcriptional analysis of adipose tissue during development reveals depot-specific responsiveness to maternal dietary supplementation
Source: Sci Rep. 2018 Jun 25;8:9628. doi: 10.1038/s41598-018-27376-3 (PMC6018169; doi:10.1038/s41598-018-27376-3)
Supplement: Supplementary file 1 — Supplementary Figures and Information [file 41598_2018_27376_MOESM1_ESM.docx]

**Transcriptional analysis of adipose tissue during development reveals depot-specific responsiveness to maternal dietary supplementation**

Authors: Hernan P Fainberg, Mark Birtwistle, Reham Alagal, Ahmad Alhaddad, Mark Pope, Graeme Davies, Rachel Woods, Castellanos Marcos, May Sean T, Catharine A. Ortori, David A. Barrett, Viv Perry, Frank Wiens, Bernd Stahl, Eline van der Beek, Harold Sacks, Helen Budge, and Michael E Symonds

**Supplement figures and supplementary legends**

Figure S1. Heat maps and principal component analysis of maternal milk fatty acid profiles

A) Heat maps and dendrograms visualizing the hierarchical clustering results (average linkage, Euclidean distance metric) found in the milk from control and diet intervention groups. Rows correspond to different fatty acids and coloured top rectangles indicate the corresponding group. The fatty acid mole percentiles were transformed to a z-score and specified by a colour code, with red representing high percentiles and blue low percentiles. B) Principal component analysis (PCA) plots represent all milk sample analysed in this study. The pink light circles are representing the control group and the dark pink rectangles represent the intervention group.


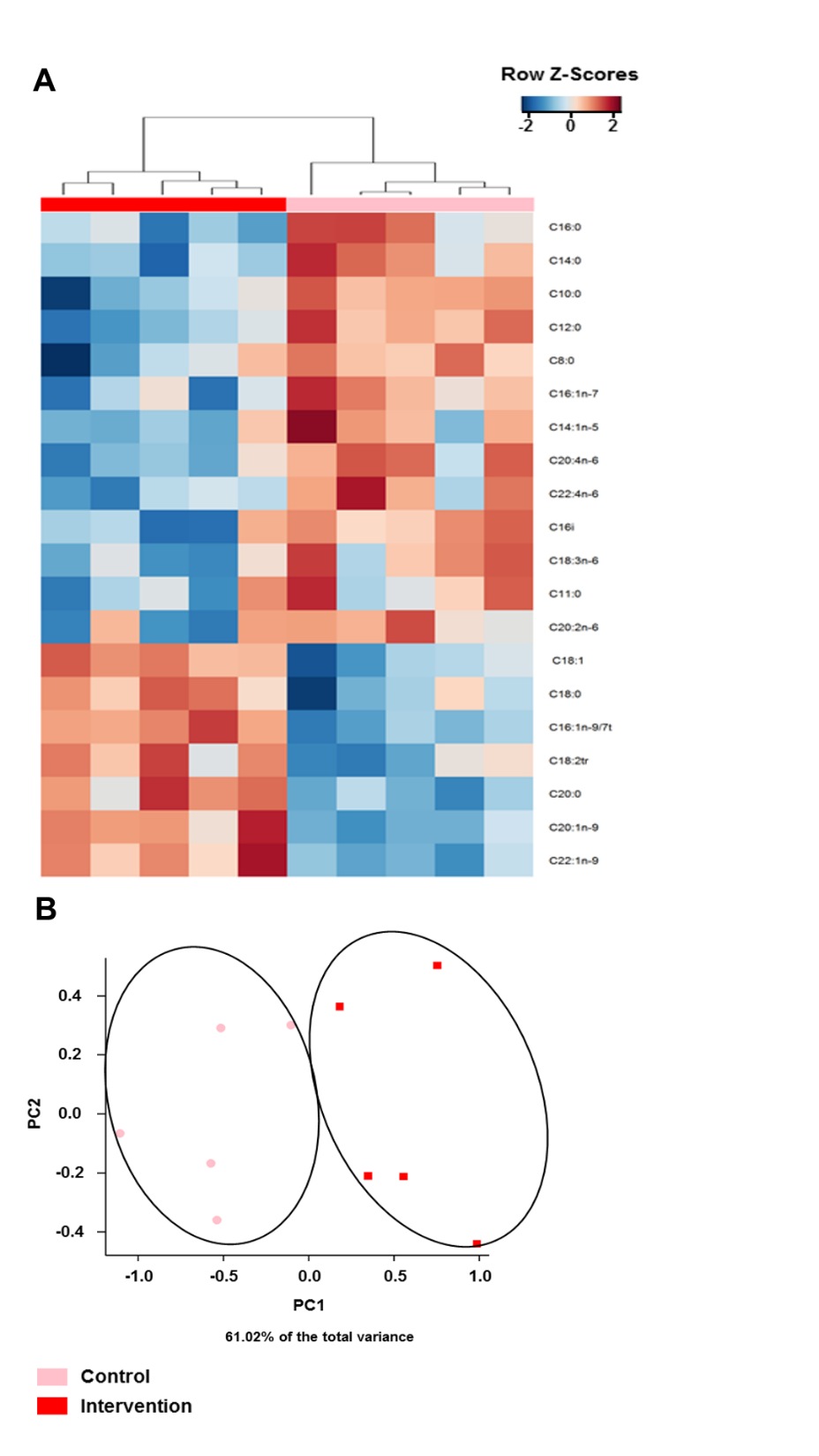


Figure S2. Comparison of gene expression analysis in perirenal adipose tissue between control and diet intervention offspring groups.

A) Heat map and unsupervised hierarchical clustering dendrogram for the top 100 differentially expressed gene transcript comparisons identified in microarray analyses of perirenal adipose tissue samples from control (dark yellow, n = 5), and maternal diet intervention (brown, n =5) offspring (average linkage, Euclidean distance metric; eBayes moderated t statistics, q < 0.05). Gene expression was transformed to a Z score. Blue indicates an increase and red a decrease in gene expression by age. B) Principal component analysis (PCA) of gene expression patterns for individual genomic interactions between both groups of offspring.


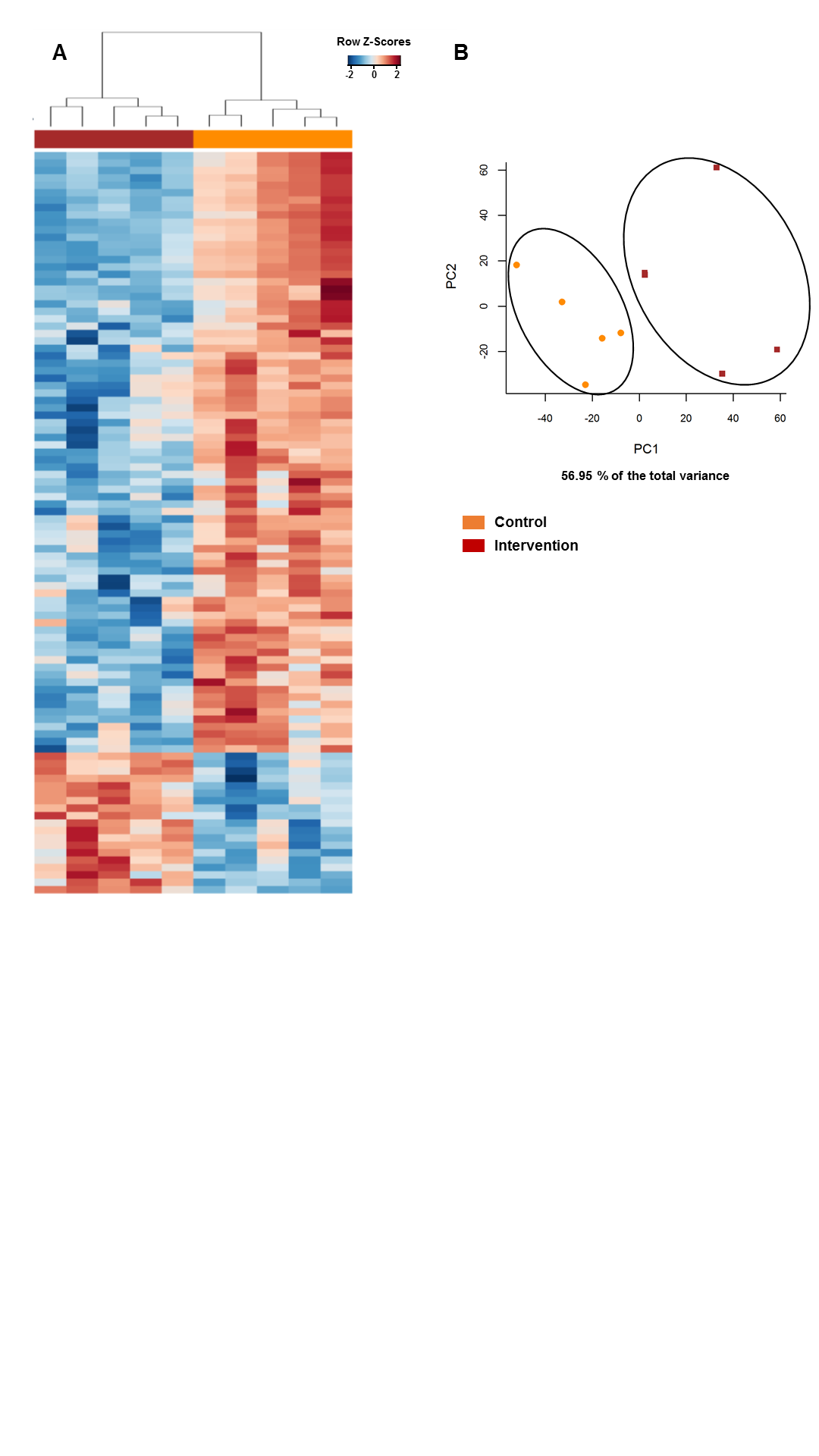


Original gels for the representative blots shown in Figure 1b


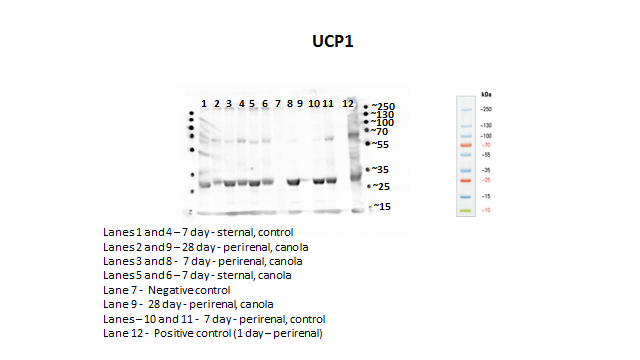


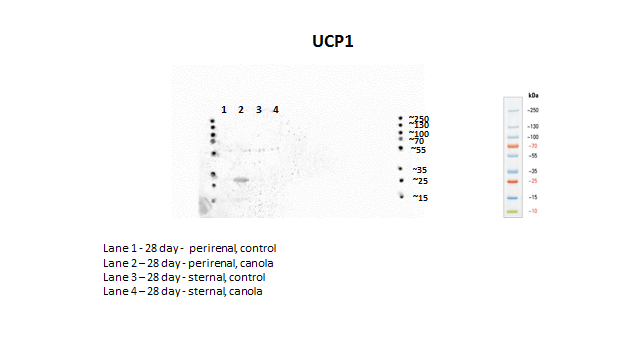


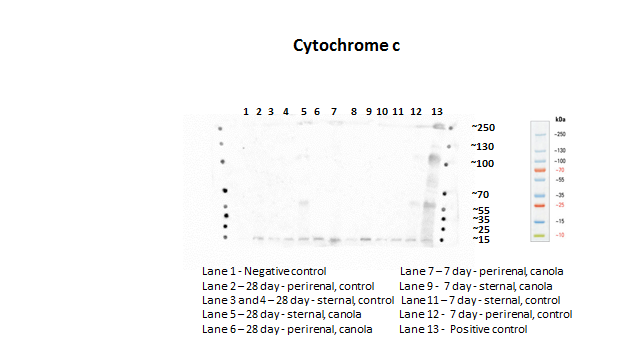


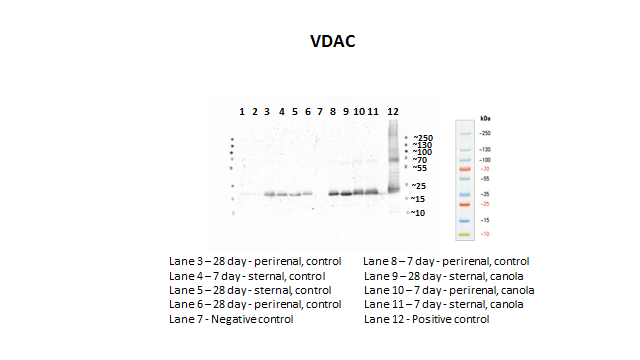


Supplement Dataset 1. Statistical results of gene expression of five adipose tissue depots at 7 days of age

Supplement Dataset 2. Statistical results of gene expression of five adipose tissue depots at 28 days of age

Supplement Dataset 3. Network characteristics of the five adipose tissue depots at 7 days of age gene co-expression modules

Supplement Dataset 4. Network characteristics of the five adipose tissue depots at 28 days of age gene co-expression modules

Supplement Dataset 5. Gene ontogeny results of each module from the 7 days of age gene network

Supplement Dataset 6. Gene ontogeny results of each module from the 28 days of age gene network

Supplement Dataset 7.Complete statistical analysis of maternal milk fatty acid profiles

Supplement Dataset 8.Complete statistical analysis of the offspring perirenal adipose tissue lipid profile at 28 days of age

Supplement Dataset 9. Statistical results of gene expression of the offspring (control vs maternal diet intervention) perirenal adipose at 28 days of age
